# Impact of Microplastics and Nanoplastics on Livestock Health: An Emerging Risk for Reproductive Efficiency

**DOI:** 10.3390/ani13071132

**Published:** 2023-03-23

**Authors:** Susy Urli, Francesca Corte Pause, Martina Crociati, Anja Baufeld, Maurizio Monaci, Giuseppe Stradaioli

**Affiliations:** 1 Department of Agricultural, Food, Environmental and Animal Sciences, University of Udine, Via Delle Scienze 206, 33100 Udine, Italy; susy.urli@uniud.it (S.U.); francesca.cortepause@uniud.it (F.C.P.); 2 Department of Veterinary Medicine, University of Perugia, Via S. Costanzo 4, 06126 Perugia, Italy; maurizio.monaci@unipg.it; 3 Centre for Perinatal and Reproductive Medicine, University of Perugia, 06129 Perugia, Italy; 4 Research Institute for Farm Animal Biology (FBN), Wilhelm-Stahl-Allee 2, 18196 Dummerstorf, Germany; baufeld@fbn-dummerstorf.de

**Keywords:** microplastics, nanoplastics, reproductive system, health, bovine, cow, cattle, BPA, granulosa cells, steroid hormone

## Abstract

**Simple Summary:**

Due to its multiple properties, such as stability, hardness and economic prices, the application of plastics has gradually increased, becoming essential in every industry. Since 1950, the worldwide plastic distribution has progressively created a serious pollution issue caused by difficulties in proper recycling, which has led to the presence of plastic fragments, called microplastics and nanoplastics (MPs/NPs), in the environment. The majority of the research has focused on the aquatic pollution, while studies regarding soil contamination are still poor, with the necessity to better understand how MPs/NPs can enter the food chain and reach humans passing through both crops and animals. Therefore, there is a need for evaluation, and the present work will provide an overview of the sources and distribution of MPs/NPs in farms; different mammalian exposure (digestion, inhalation and dermal contact) and associated risks and health problems caused by these fragments. In particular, this review aims to provide information on the effects, mainly from additives (such as Bisphenol A-BPA), on livestock reproduction and fertility.

**Abstract:**

Pollution due to microplastics and nanoplastics is one of the major environmental issues of the last decade and represents a growing threat to human and animal health. In aquatic species, there is a large amount of information regarding the perturbation of marine organisms; instead, there are only a few studies focusing on the pathophysiological consequences of an acute and chronic exposure to micro- and nanoplastics in mammalian systems, especially on the reproductive system. There are several studies that have described the damage caused by plastic particles, including oxidative stress, apoptosis, inflammatory response, dysregulation of the endocrine system and accumulation in various organs. In addition to this, microplastics have recently been found to influence the evolution of microbial communities and increase the gene exchange, including antibiotic and metal resistance genes. Special attention must be paid to farm animals, because they produce food such as milk, eggs and meat, with the consequent risk of biological amplification along the food chain. The results of several studies indicate that there is an accumulation of microplastics and nanoplastics in human and animal tissues, with several negative effects, but all the effects in the body have not been ascertained, especially considering the long-term consequences. This review provides an overview of the possible adverse effects of the exposure of livestock to micro- and nanoplastics and assesses the potential risks for the disruption of reproductive physiological functions.

## 1. Introduction

Plastics have been widely used in production and life ever since their invention due to their remarkable properties of durability, lightness, stability and low cost. Plastic products have revolutionized our social life to such an extent that experts speak about the “Plastic Age” [1] or “Plasticene” [2]. The production of plastic per year has increased tremendously, as the global plastic production reached 390 million tons in 2021 compared to only 2 million tons produced in 1950 [3]. The demand for plastics in Europe reached 50.7 million tons, with Germany in the lead (24.2%) and Italy in second (13.8%). One of the largest end use markets is the packaging and building/construction industries. Interestingly, both sectors have the most different product life cycles [4]. While plastics in the building and construction sector are functional for 35 years, some plastics, especially in the packaging industry, might have very short lifetimes of 6 months or are single-use only, thus contributing to the immense waste management issue. It is noteworthy that the COVID-19 pandemic has increased the plastic use and environmental contamination by plastic as a result of the common use of masks, gloves and other plastic consumables. This has enormous effects on daily life not only regarding humans but also other animals. The physicochemical characteristics and the mechanical and technological properties of plastics have led to an increased worldwide distribution. The main characteristics of plastics are hardness, resistance to stress and impact, elasticity, machinability and economical cost. Plastic is a macromolecular material composed of polymers of different lengths. The most common compounds used to make plastics are polyethylene (PE), polypropylene (PP), polystyrene (PS), polyethylene terephthalate (PET) and polyvinyl chloride (PVC). Furthermore, various additives such as plasticizers, flame retardants, stabilizers, colorants, antistatic agents, lubricants, slip agents, curing agents, foaming agents and biocides are used to enhance their performance [5]. The results from several studies indicated that these additives pose a greater risk to physiological functions than plastics. Commonly used additives are phthalate esters and bisphenol A (BPA). Phthalate esters serve to make PVC more flexible and softer [6], and BPA is used because of its translucent property, to increase the mechanical and thermal resistance [7]. In general, plastic particles can be divided into two categories: primary particles, which are intentionally produced by the industry for various purposes (pellets used to make plastic products, abrasive microbeads or personal health care products), while secondary particles are generated when there is the disintegration or abrasion of materials or waste released into the environment (washing synthetic clothes, tire abrasion, etc.) [8]. The exposure of plastic waste to physical, mechanical, chemical and biological processes such as fragmentation, weathering, hydrolysis, UV radiation and biodegradation leads to the production of microplastics (<5 mm, MPs) and nanoplastics (<0.1 µm, NPs). Plastic residues persist in the environment, especially in marine and aquatic ecosystems; it is estimated that more than 68% of these residues in the oceans originate from the fragmentation of waste that is not disposed of or improperly recycled. Not to be underestimated are the biodegradable plastics, which presence in the environment is increasing due to incomplete biodegradability and increasing use [9,10]. The ecotoxicological effects of MPs/NPs on marine phytoplanktons and zooplanktons, invertebrates and plants are well documented, while ingestion and accumulation from marine prey, leading to transfer to the predators, also occur [11,12]. The distribution of plastics is ubiquitous in the environment and includes atmosphere, soil and water; this likely represents a potential entry of microplastics into the food chain and, therefore, a concern for human and animal health. The results from a study of plastic particles on agricultural farmland in Germany are indicative of the importance of the soil cycle, as conventionally treated farmland had greater MP contamination compared to aquatic ecosystems [13]. The three main routes by which microplastics and nanoplastics can enter the human and mammalian body are the (1) inhalation of airborne plastic particles originating from synthetic textiles and polluted outdoor air, (2) ingestion of contaminated food and water supplies and (3) skin contact, with these plastic particles passing through the skin barrier [9]. In addition, due to their chemical–physical properties, these materials may facilitate the binding and transport of chemical contaminants (e.g., antibiotics and heavy metals) and microbial agents (e.g., bacteria), thus increasing their impact on the environment and on human beings and animal health [14]. Several types of toxic chemicals have been reported to be associated with MPs, most of which are either heavy metals (e.g., arsenic, zinc, copper, cadmium, lead and chromium); persistent organic pollutants (POPs); polychlorinated biphenyls (PCBs); polycyclic aromatic hydrocarbons (PAHs) and organic pesticides [15]. In addition, several microorganisms are able to bind to MPs, such as fungi, diatoms, algae and, most commonly, bacteria [14]. All microbial and chemical associations with MPs depend on various factors such as MP type and size, PH, salinity, plastic aging effect and polymer crystallinity [16]. Microplastics affect the evolution of microbial communities and increase gene exchanges, including antibiotic resistance genes (ARGs). There are no published findings on the abundance and diversity of antibiotic resistance genes in bacterial taxa in the marine plastic environment, although seawater has been identified as a global reservoir for ARGs and for metal resistance genes (MRGs) [17]. In recent decades, the emphasis has been placed on the effects of plasticizers and additives, while the direct effect of plastics has only recently been studied in more detail. Several studies have attempted to gain a better understanding of the mechanisms for the toxicity of MPs/NPs in mammalian cells, and there is evidence that these plastic particles induce damage such as oxidative stress, inflammation, apoptosis and dysregulation of the endocrine system. However, there is very little research on the amount, types and toxicities of nanoplastics and their effects on livestock health. In this review, therefore, a thorough look at the epidemiology of nanoplastics and microplastics in the food-producing animal production system, at the effects on the physiological system and degradation within the environment, quantities of toxicity, contamination and effects on animal health, with a focus on the reproduction, will be given.

## 2. Methodology 

This review was prepared from findings after conducting a search using PubMed, Web of Science, ScienceDirect, Scopus, Google Scholar and Google with the following keywords and strings: “Microplastics”, “Nanoplastics”, “Reproductive system”, “Health”, “bovine”, “cow”, “cattle”, “BPA”, “Granulosa cells”, “Steroid hormone”, “Endocrine disrupting chemicals”, “Exposure”, “Migration” and combinations. The last accession to the online databases was conducted in January 2023. For information to be included in this review, the manuscripts had to meet the following criteria: to be related to mammals and to have a focus on the reproductive system and the disruption that plastic particles and additives may cause.

Additionally, references and citations from relevant publications were also manually screened to gather further information. The search results were then reviewed and the information analyzed, categorized and presented in sections to effectively address the scope of this review.

## 3. Resources and Distribution of Microplastics in Farms

Plastics are ubiquitous in many industrial and urban sectors, including agricultural production, building and construction, transportation, packaging, electronics and automotive manufacturing. Additionally, plastics cause “visible pollution” through contributing to a large volume of total municipal solid waste and “invisible pollution”, which poses a major threat to air, oceans, soil, livestock, wildlife and marine life [18]. A large amount of information on plastic particle contamination in the aquatic environment is available, but there is much less information regarding the transfer of these agents to soils. There are many pathways for plastic particles to enter a soil environment. These include the fragmentation of larger plastics such as agricultural plastic mulch film used in horticultural and agricultural processes. Another pathway includes atmospheric or airborne deposition, especially from uncovered or mismanaged landfills or urban litter. Plastics can also enter the soil through the irrigation of agricultural land with contaminated water or road runoff (e.g., tire abrasion). Other potential pathways include the use of plastic-coated fertilizers and the application of biosolids (e.g., sewage sludge from wastewater treatment plants) [19]. Biosolids and plastic mulch films are the most prevalent plastic contaminants in soil. In several countries, biosolids continue to be extensively applied to agricultural soils to improve their physical properties and maintain productivity [20]. Biosolids retain and accumulate as much as 99% of the plastic particles introduced via the influent, with an increasing risk of accumulation in soils after the repeated or long-term application of treated sludge. Generally, sewage sludge must undergo treatments such as aerobic or anaerobic digestion, composting, alkaline stabilization and thermal drying before land applications to reduce the pathogen load, control odors, reduce the vector attractiveness and inactivate heavy metals. Limited data are currently available on whether these treatment processes remove plastic particles from biosolids before land applications [19]. Instead, agricultural plastic mulch films are used to improve the efficiency of water retention, pesticide and nutrient use. Thermal insulation during the early planting or harvesting of crops may reduce soil erosion, suppress weed growth and reduce crop disease burden [21]. Zhu et al. [22] reported that plastic films are usually thin, about 10–30 µm, which makes removal from the field after the growing season very difficult and recycling less feasible. For long-term applications, residual plastic mulch films in fields may slowly fragment into smaller particles through the actions of soil tillage, UV radiation, water and wind [23].

**Figure 1:**
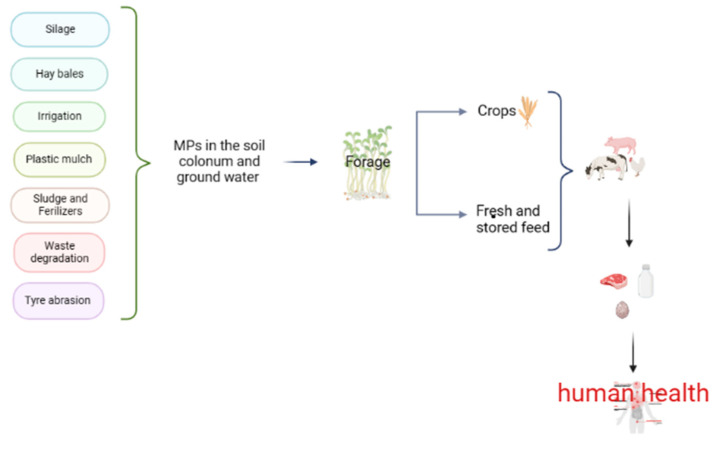
A schematic diagram of the sources and fate of plastic particles in the soil. Silage covers, strings of the attached when baling hay, irrigation with water contaminated by MPs, plastic mulch, sludge and fertilizers, municipal waste degradation, tire abrasion and roadside litter affect the structure, fertility, nutrients and microbes of the soil. Land is used to produce feed for food-producing animals, which can be consumed fresh or stored. All of these sources increase the risk to human health through the ingestion of MPs from milk, meat and eggs.

In addition to the risk of soil contamination from agricultural practices, the atmosphere is an important source for the plastic contamination of soil. Crude particles with a diameter of less than 2.5 µm enter the atmosphere through mechanical processes such as dust resulting from winds, thus increasing the risk of soil deposition. In addition to these previously described pathways, the use of manure from biowaste composting, tire abrasion, film coating of agronomic seeds, roadside littering (especially close to agricultural land) and illegal dumping of waste all contribute as sources of plastic particles in soils [19]. Concerning livestock farms, the risk of animals ingesting or simply coming into contact with microplastics cannot be excluded, as well as the presence and accumulation of these particles in animal products such as meat, milk and eggs (Figure 1) [24]. Plastic particles from contaminated soil can be ingested by animals and excreted in feces, leading to further dispersal of the pollutant [21]. As previously described in this manuscript, many soils are contaminated with microplastics as a result of both agronomic techniques and human negligence by leaving litter on fields where crops are later grown. MPs can be taken up through plant roots, especially nano-sized particles, and transported to edible parts of the plant [25]. In North America, 44,000 to 300,000 tons of MPs are deposited on agricultural soils annually [26], while an estimate of 63,000–430,000 tons has been described for European farmlands [27].

In addition to the possibility that plants that are subsequently eaten by animals absorb plastic particles, we must also consider the techniques used to store food. Forage is the basis of the diet of dairy and beef cattle and is covered with a plastic film for preservation [28]. Hay bales are also wrapped with mesh or twine to maintain their shape, both of which contribute to the use of plastic in feed preservation practices for food-producing animals. This practice, therefore, increases the risk of the migration of MPs or additives from feed packaging into the feed. An example is the reporting by Wang et al. [29], where the presence of bisphenol products (BPs) was observed in animal feed. The BPs are found in PP and PE packaging and can migrate into the solid feed of cows, with the risk of being transferred into their milk, as reported by Russo et al. [30]. 

The results from another study by Zhou et al. [31] indicated that nonpackaged fresh meats, such as pork, chicken, beef and mutton, were contaminated with BPs, thus suggesting that an additional contamination pathway other than migration from food packaging may be possible. Due to the highly lipophilic nature of BPs, bioaccumulation in animals and derived animal products (such as eggs, milk, and meat) may occur as a result of exposure to BPs in feed [32].

Considering both humans and also other livestock species such as pigs and poultry, there should be a focus on the presence of MPs in fishmeal and fish oil, which are widely used as a nutritional source in food-producing animal feed [15,33].

There is also a prevalence of MPs in drinking water [25]. Information, including a recent World Health Organization’s (WHO) report on “Microplastic in drinking water” [34], indicates that there is not yet proof of harm and calls for more research to be conducted so there can be a greater understanding of the potential detrimental effects of microplastics in drinking water [35]. Kosuth et al. [36] tested tap water for human consumption from 159 global sources, and 81% contained microplastic particles less than 5 mm in diameter. Plastics in the soil threaten food safety and, therefore, the health of all organisms, and the environment may be affected in similar ways as the plastic pollution of the oceans.

## 4. Mammalian Exposure to Micro- and Nanoplastics

There are three primary routes by which microplastics and nanoplastics can enter the animal body: ingestion, inhalation and skin contact. The results from many studies that are subsequently addressed in this manuscript indicate that the amount of absorption after exposure is correlated with the size and concentration of the plastic particles, as well as the tissue and cell types.

### 4.1. Mammalian Exposure to Microplastics through Ingestion and Drinking Water

Animals ingest microplastics and nanoplastics because of their presence in different feeds and forages. Firstly, microplastics are ubiquitous in surface water, groundwater and wastewater [8,37], with different types of plastics such as fragments, fibers, films, etc. present in feed sources [38]. Animals and humans drink water contaminated with MPs, and microplastics are present in water used for the irrigation of fields [32,39]. In a previously mentioned article, considerable elaboration on how arable land is contaminated by MPs was provided. These nanoparticles can be absorbed via plant roots and transported through the xylem pathway to edible parts [40]. In cultivated plants, this may also mean that plastics can be transferred to the part of the plant that is intended for human or animal consumption and thus enter the food chain [41]. In some intensively cultivated areas of Europe, where ruminants graze after the harvesting of grains, the ingestion of plastic fragments occurs [21]. In developing countries, such as Ethiopia and India, however, the issue of plastic waste is even more widespread, because many animals, including livestock, are not maintained in confined areas and feed on garbage. When ingested, plastics slowly release chemicals in the rumen, which can enter the systemic bloodstream and contaminate milk and meat products and the food chain. These chemicals have adverse effects on human health [18,42].

Another risk is represented by the migration of additives or MPs from plastic packaging into solid animal feed. Wang et al. [29] confirmed this potential transfer route, but there are no reports investigating the effects of plastic particles after passage into the gastrointestinal tract of food-producing animals. One potential scenario is that these compounds remain in the intestinal lumen or migrate across the intestinal epithelia [9]. In fish and mice, there is some information on pathological manifestations associated with the absorption of nanoplastics across the gastrointestinal wall. In mice, ingested MPs/NPs were detected in the intestine, liver and kidneys. In the gut, the plastics induced alterations such as a reduction in mucosal secretion, intestinal barrier dysfunction, inflammation and microbiota dysbiosis. In the liver, however, these particles led to inflammation and to subsequent alterations in the blood lipid profile. Additionally, the absorption and accumulation of MPs led to various types of disorders in mice [35]. Based on these pathological outcomes in mice, it will be important to understand how the ingestion of nanoplastics may also affect food-producing animals. 

Huerta Lwanga et al. [43] reported a possible trophic transfer of MPs from home gardens to earthworms and chickens. In chickens, MPs were recovered from the gizzard lumen and feces. In addition, it is noteworthy that there were different MP particle sizes transported through the digestive system, from the chicken crop (>5000 µm) to the gizzard (<5000 µm) and into feces (100 to 1000 µm). It has been postulated that plastic ingestion led to a reduction in gizzard volume, which, in turn, decreased the foraging time and, hence, growth [44]. Zhang et al. [45], however, reported an estimate of MP intake ranging from 3 to 677 mg/week for domestic animals. Campanale et al. [2] reported that humans ingest about 80 g/day of microplastics through plants (fruits and vegetables) that accumulate MPs through plant uptake from polluted soil. There have been no specific studies in cattle, but these previous findings in humans suggest that there is another route of MP intake in herbivores [25].

### 4.2. Mammalian Exposure to Microplastics through Inhalation

The second most likely route of exposure of mammals to MPs/NPs is through inhalation. Minute particles of plastic may be suspended in the air; they mainly originate from synthetic textiles, but also, the inhalation of dried wastewater fertilizer or atmospheric fallout occurs [46]. Air contaminated with MPs/NPs comes into direct contact with the respiratory tract, affecting the mucus layer, periciliary layer, ciliated cells, non-ciliated secretory cells and basal cells. Considering the extremely fine structure of the alveolar surface, NPs may penetrate this tissue, thus entering the bloodstream and, subsequently, other body tissues [9]. In a cell culture of human alveolar epithelial cells, there were cytotoxic effects, oxidative stress responses and inflammatory responses against MPs. Generally, a rough estimate of human exposure to MPs by inhalation and dust ingestion is in the order of a few milligrams per day [47].

### 4.3. Mammalian Exposure to Microplastics through Skin Contact

Another entrance pathway of MPs/NPs could be transdermal, more specifically by contact or injection. Plastic particles can pass through the skin with the use of health and beauty products (only in humans) or contact with contaminated water. The point of access for MPs/NPs could be the stratum corneum, but they could also transfer via the sweat glands, skin wounds or hair follicles [9]. The outermost layer of the skin, the stratum corneum, forms a natural barrier, making it unlikely that molecules will penetrate this tissue layer if in an intact state. Alvarez-Roman et al. [48], performed a study on the penetration of polystyrene particles ranging from 20 to 200 nm in diameter into the stratum corneum of pigs. Many 20 nm polystyrene NPs concentrated in the hair follicles of these pigs, even though the particles were not transferred into the inner layers. Thus, the results from this latter study indicate that there is only a superficial skin penetration of MPs/NPs. However, it cannot be excluded that these particles may enter the systemic circulation by means of plastic-based intravenous catheters, syringes and other drug delivery systems [49].

There has been elaboration on the current knowledge in the present article regarding the different entry routes of small plastic particles; however, the possible deposition and effects of these compounds in animals have yet to be resolved. 

One thing is certain: once these compounds enter the body, there is not a ready clearance from the tissues. Rather, there is a presence of NPs in the blood and consequent transport via the blood circulation to all the tissues of the body [50].

## 5. Risks of Exposure to Microplastics and Nanoplastics in Food-Producing Animals

The risk posed by microplastics and nanoplastics to humans and animals is physical, chemical and microbiological nature. Physical risks are due to the small sizes of MPs/NPs that can cross biological barriers such as the skin, gut, hemato–encephalic, testicular and even placental tissues and cause direct damage. The chemical risks are due to the presence of persistent additives or contaminants that are potentially hazardous, while the microbiological risks are related to microorganisms adhering to the MP surface [51].

The exposure of animals to MPs results in inflammation; cytotoxicity (e.g., oxidative stress, cells damage, cell viability and altered membrane function); genotoxicity (through oxidative damage) and immunotoxicity [52]. Many of the toxic effects of MPs are intricately interconnected, as perturbation of one process may trigger a cascade of other toxicological responses [47]. The toxicity, translocation and accumulation of MPs depend on their size, shape, dose, surface functionalization and charge, as well as hydrophobicity. There is convincing evidence that MPs accumulate in tissues. The results from many studies [8,9,35] are indicative that inflammation, oxidative stress, apoptosis, necrosis and immune responses occur because of the accumulation of MPs/NPs in human and animal tissues. 

**Figure 2:**
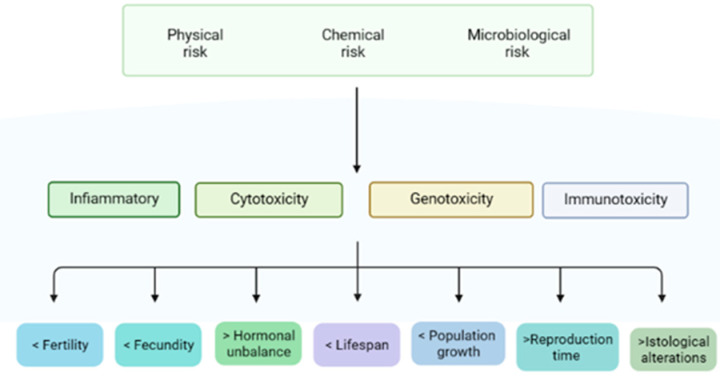
Risks, effects and consequences of MPs/NPs on reproduction.

Particles < 100 μm in diameter can cross cell membranes, and particles < 20 μm can be efficiently translocated to various organs. Kannan and Vimalkumar [47] reported the accumulation of PVC particles in different species (e.g., in pigs) in the 1970s. There is also evidence that the majority of the larger ingested particles are excreted through feces. Smaller particles, however, can be absorbed systemically and may partially pass through tissue barriers. The blood–brain barrier, as well as the placental barrier, may be crossed by particles ranging from 0.1 to 10 µm in diameter, while passage through the gastrointestinal tissue walls can occur for MPs as large as 150 µm. Presumably, plastic particles smaller than 2.5 µm can also circulate systemically in the organism by endocytosis. Ragusa et al. [53] analyzed six human placentae from Rome (Italy), which were evaluated using the Raman microspectroscopy technique; in four out of the six specimens, 12 MP fragments (5–10 µm) were observed. Interestingly, all the MPs were pigmented, suggesting their origin from coatings, paints or personal care products. Furthermore, the particles were not only in the maternal side but also in the fetal side of the placenta and in the chorioamniotic membrane, thus highlighting a potential risk to the fetus. The authors hypothesized that the plastic particles in placentae could interfere with major cellular pathways that regulate immune system functions, growth factor signaling and several other systems. Wick et al. [54] also reported that polystyrene particles 240 nm in size can cross the placental barrier through diffusion or binding to cellular transport proteins. The accumulation of MPs primarily occurs in the liver, kidneys, gut [25], stomach, small intestine and mesenteric lymph nodes [49]. Fournier et al. [55] administered 0.02 µm polystyrene particles to late-gestation female rats and observed that the transfer of these particles to fetal tissues, including the liver, lungs, heart, kidneys and brain, occurred. Lou et al. [56] also reported that, after maternal exposure to polystyrene microplastics, the resulting offspring had various metabolic disorders, such as an alteration of the serum triglyceride and cholesterol concentrations. This is indicative of the potential risks of the microplastics to the reproductive tract, as well as to the fetus, in all species (Figure 2).

Additionally, the toxicological risk of microplastics and nanoplastics is increased due to the large amount of additives used in the production of these polymers, as emphasized in the introductory section of the manuscript. The most common and harmful additives are Bisphenol A (4,4’-(propane-2,2-diyl) diphenol) and phthalate esters, including DEHP (Bis(2-ethylhexyl) phthalate) and MEHP (mono- (2-ethylhexyl) phthalate) [9]. These chemicals are cytotoxic and can also behave as endocrine disruptors (EDCs); therefore, alterations of the reproductive physiology of animals may occur as a result of the hormonal activity of these compounds [57]. In fact, EDCs are considered more harmful than MPs, since these compounds are responsible for the induction of cancer [25], mutations of DNA and toxic reproductive effects. Moreover, these chemicals are recalcitrant in the environment, can accumulate in the food chain and bodies and show harmful proprieties such as hormone disruptors [2]. It has been demonstrated that exposure of laboratory animals to MPs and their additives leads to the disruption of adipogenesis and lipid metabolism through the activation of peroxisome proliferation-activated receptors (PPARs: master regulators of adipogenesis), suggesting that MP exposure may be associated with the increasing prevalence of obesity globally [47].

Another issue related to microplastics is represented by the microbiological risk, because several microorganisms (MOs) such as fungi, diatoms, algae and bacteria are able to adhere to MPs [14]. This ability can be attributed to different electrostatic charges (negative charge of MOs and positive charge of MPs). MPs have a biofilm surface that protects and supports MOs (especially bacteria), promoting microbial multiplication and spreading to body tissues. Consequently, these bacteria absorbed by plastics are exposed to contaminants, such as antibiotics and metals; this phenomenon may also significantly contribute to modifying their antibiotic resistance through co-selection. Yang et al. [17] reported the multidrug resistance genes and multi-metal resistance genes were the main classes of genes detected in plastic-associated microbiota. The most important source of antibiotic resistance genes (ARGs) is hospital wastewater, which is treated in domestic wastewater treatment plants before being mixed with the receiving water; other sources include waste and runoff from animal husbandry [58]. Further studies, however, are needed to better understand the actions of MPs in the dissemination/spread of ARGs in different environments, such as water, soil and air.

In addition, persistent organic pollutants and polycyclic aromatic hydrocarbons (PAHs) bind to MPs, which could lead to endocrine disruption and possibly cell death or mutagenesis [59]. Both phthalates and persistent organic pollutants have been found in the egg yolk of a sea turtle (*Caretta caretta*), resulting in altered embryonic development and in failure of egg hatching [60,61]. This illustrates how MPs and their additives, as well as the substances/MOs that may adhere of their surface, could be harmful both to animals and their offspring.

## 6. Effects of Microplastics and Nanoplastics on Reproduction

The exposure of MPs/NPs may trigger toxicity pathways, including the exacerbation of inflammation and oxidative stress (OS). After being absorbed, MPs/NPs may have actions locally or be transported to the bloodstream and, after the translocation, may reach all organs and tissues, including the gonads (see [12] for a detailed description of the translocation routes). The NPs can also accumulate in several reproductive tissues, thus inducing reproductive dysfunction(s). Reproductive alterations are mainly mediated by oxidative stress and are also associated with the upregulation of prooxidant mediators (reactive oxygen species, lipids and DNA oxidation); cell death; proinflammatory molecular pathways and cytokines and the inhibition of enzymatic and nonenzymatic antioxidant defense mechanisms. 

In the female mouse reproductive system, the major microstructural abnormalities identified consisted of dilatation of the oviducts, presence of ovarian cysts and increased number of corpora lutea, decreased thickness of the granulosa layer in secondary follicles, reduced number of growing follicles, greater accumulation of ovarian collagen and fibronectin and apoptosis of granulosa cells [62]. Some of these effects have also been observed in rats, as indicated by lesser serum anti-Mullerian hormone concentrations as a marker for follicle reserves [63]. Furthermore, MPs/NPs increase fibrotic processes in the ovaries and in granulosa cells by increasing the levels of ROS (reactive oxygen species) and MDA (malondialdehyde) and decreasing the activities of antioxidant enzymes, including SOD (superoxide dismutase), CAT (catalase) and GPx (glutathione peroxidase) [12]. The results from in vivo studies in rats indicated that the accumulation of ROS could lead to GC apoptosis and to follicular atresia, which may be the causal factor for infertility as a result of anovulation [63].

In mice and rat male reproductive systems, microplastics and nanoplastics detection in the testes was associated with multiple microstructural alterations, including testicular atrophy, incomplete spermatogenesis, disorganization or disruption, as well as with increased permeability of the blood–testis barrier [57]. Concerning the male gametes, greater amounts of sperm abnormalities have been observed in association with the presence of MPs and NPs; the major defects consisted of head and tail alterations, as well as acrosome loss. Additionally, other seminal characteristics were affected and resulted in a lesser sperm motility or immobility, apoptosis and an overall lower sperm count [62,64]. Deng et al. [65] reported an increase in SOD and MDA contents in testes when exposed to MPs, suggesting the involvement of oxidative stress pathways in the disruption of testicular functions.

Concerning embryonic development, it has been suggested [12] that MPs/NPs induce germ cell abnormalities by altering the fluidity of the membranes that are in contact with gametes, with the MPs not entering the embryo but adhering to the surface of the chorion and reducing the exchange of oxygen, followed by embryonic physiological disruption. Yin et al. [66], however, reported that NPs could be transported into the embryo and accumulate in the yolk sac, leading to alterations in nutrient absorption. All the mentioned studies referred to aquatic organisms.

The results from several studies in women indicate there is a presence of MPs in the fetal and maternal placenta and chorionic membranes, which might be harmful for a pregnancy, because the placenta supports fetal development and provides an interface between the internal and external environment [12,53,54]. The results from other studies implicate polystyrene MPs as a cause of alterations in the sex ratio and weight of offspring in mice, as well as a dysfunction of the lipid and amino acid metabolisms; therefore, there is the potential for interfering with the physiological functions of future generations [62].

Microplastics and nanoplastics induce the proinflammatory and prooxidant processes, as well as the imbalance in reproductive hormone concentrations in male and female animals. Considering the inflammatory effectors, the plastic particles had effects in upregulating the abundance of TNF-α (tumor necrosis factor), interleukin IL-1ß, IL-6, IL-8 and the apoptotic factor caspase-3 [49,67]. The hormonal panel showed a consistent downregulation of T4 (testosterone), LH (luteinizing hormone), FSH (follicle-stimulating hormone) and AMH (anti-Mullerian hormone) concentrations [57]. After exposure of mice to polystyrene MPs/NPs, the concentrations of FSH, LH and T4 decreased and estradiol level increased in the serum of males, while, in females, the observed hormone changes were the opposite [62].

Considered together, the results from all these reports highlighted the negative effects of plastics on reproductive tissues and functions, which may compromise the reproductive efficiency in humans and animals.

## 7. Effects of Bisphenol A and Other Additives on Fertility and Reproductive System on Livestock

In addition, to plastic particles, three plastic additives (Bisphenol A, phthalates and polychlorinated biphenyl 153) have been identified as causing infertility. These are defined as endocrine-disrupting chemicals (EDCs), as they are able to interfere with the endocrine system, thus mimicking hormonal active agents. The trend for decreased the fertility rate and reproductive failure in farm animals may be a consequence of acute or long-term exposure to EDCs [68,69,70,71,72,73].

Bisphenol A (BPA was tested for estrogen activity in the early 1930s; it is a xenoestrogen with estrogen-mimicking, hormone-like properties. The BPA compound acts as an estrogen antagonist. It can bind to estrogen (ERs) and androgen (AR) receptors, thus interfering with steroidogenesis in Leydig cells, including 17α-hydroxylase/17,20 lyase and aromatase functions, interfering with LH receptor-ligand binding [74]. Increasing evidence that BPA has an effect on both female and male fertility is available. Concerns regarding BPA have led to the use of alternatives, one of which is Bisphenol S (BPS); the latter has been determined to be a “regrettable substitution”, since BPS showed similar or even worse detrimental effects than BPA [75].

Another additive that has actions as an EDC and therefore interferes with fertility is DEHP (Bis(2-ethylhexyl) phthalate). It interacts with estrogen metabolism by suppressing the enzyme aromatase, which is necessary for the conversion of testosterone to estradiol and has an important role in brain sexual differentiation [76]. The DEHP compound may also induce abnormalities in the male reproductive tract.

Ding et al. [77] described the negative effects of BPA on female mouse fertility, which were due to impaired cytoskeletal dynamics in the oocyte, induction of oxidative stress, increased DNA damage and epigenetic alterations in oocytes. The BPA compounds can affect the follicular, ovarian and the hypothalamic systems, granulosa and theca cells and induce the formation of progressive proliferative lesions on the oviduct and uterus, such as atypical hyperplasia, stromal polyps and endometriosis. Lambs exposed to BPA had reduced follicular ovarian reserves with a lesser population of primordial follicles, an increase in antral atretic follicles, a greater prevalence of follicles containing multiple oocytes and reduced ovarian weights [74].

Fujimoto et al. [78] observed an association between a greater concentration of BPA in the serum of women and decreased likelihood of mature oocytes. Saleh et al. [79] also reported that BPA increased apoptotic gene expression in bovine oocytes. Both BPA and BPS (such as BPA) disrupt oocytes-secreted proteins (GDF9 and CX37), damage the gap junctional intercellular communication of COCs (cumulus–oophore complexes) [80] and impair the prophase I-to-MII transition in oocytes [81]. In addition, BPS has effects on the relative abundance of maternal mRNA, while BPS exposure induced changes in the protein secretion, distribution of estrogen receptors α and ß and of aromatase in oocytes [75].

Relatively greater concentrations of BPA were detected in the urine of infertile compared with fertile women and in those with polycystic ovary syndrome (PCOS), where an association between the BPA content and greater androgen concentrations were observed [82]. BPA has also been detected at different concentrations in the serum of pregnant and nonpregnant women, follicular fluid, fetal serum and amniotic fluid [83].

There was no BPA detected in the follicular fluid of pigs, but BPA alters the hyaluronic acid production and gene expression of cumulus cells and disrupts the spindle formation and meiosis in oocytes [75]. BPA has been found in cattle urine [84] and women’s follicular fluid at a concentration of 2.4 ± 0.8 ng/mL, respectively [85].

Concerning the hypothalamic–pituitary–gonadal axis (HPGA), BPA interferes with a gonadotropin synthesis by reducing the relative abundance of “gonadotropin mRNA, GnRHr, and Nr5al, key components of gonadotropin synthesis” [86]. Xi et al. [87] reported that estrogen production by granulosa cells of mice is affected by BPS through the disruption of HPGA, similar to the effects of BPA. Treatment with BPA resulted in an impaired reproductive capacity and delayed onset or even failure to express puberty [88]. In women undergoing in vitro fertilization, relatively greater urinary concentrations of BPA were correlated with a failure of embryo implantation; greater serum concentrations were then associated with the prevalence of abnormal embryos and premature parturition [89,90,91]. 

Additionally, the detection of BPA was associated with a reduced cleavage rate and development of embryos at the blastocyst stage and alteration in gene expression in cattle [80]. The results from several studies on rat pups produced by a dam exposed to BPA showed reduced birth weights, lower weights in males, especially before birth, and a positive correlation between maternal BPA and both weight/size of the offspring [92,93]. Other studies, such as Talpade et al. [73], have led to results indicating adverse effects of BPA in chickens (*Gallus domesticus*), such as increased embryo mortality and the malformation of reproductive organs. 

Additionally, Gao et al. [94] observed a correlation between BPA and breast and ovarian cancers and endometrial carcinoma.

Phthalate esters are also active in the female reproductive system, with DEHP affecting ovarian function, which causes decreased serum estradiol concentrations, prolonged estrous cycles and failure of ovulation and cystic progression [6]. Maternal exposure to DEHP resulted in reproductive toxicity and led to modulation in the abundance of molecules that regulate uterine function in the following generation of rats [95]. The MEHP compound (monoethylhexyl phthalate), the active metabolite of DEHP, is assumed to be able to suppress aromatase in granulosa cells through the activation of PPARs (peroxisome proliferator-activated receptors). Then, MEHP probably inhibits the meiotic maturation of oocytes in cattle [96]. 

Inconsistent with these actions, BPA binds and has functions as an androgen receptor antagonist (AR) and alters the 17α-hydroxylase/17,20 lyase and aromatase expression and LH receptor–ligand binding, thus interfering with steroidogenesis in Leydig cells [97]. 

In 98% of men with infertility problems, there is a correlation between urinary BPA and sperm count and motility [98]. Additionally, BPA alters the energy metabolism and reduces sperm storage, sperm transit time and mitochondrial activity while increasing the apoptosis of Sertoli cells, the percentage of immature sperm and sperm DNA damage, thus determining the lesser semen quality [74,99,100]. These alterations have also been found in dogs, cats and goats, while the possibility of an increased prevalence of prostate cancer has been suggested [80].

As for hormones, relatively greater BPA concentrations are associated with a reduction in testosterone and LH, leading to hypogonadotropic hypogonadism, and are associated with a greater FSH: inhibin B ratio and lesser estradiol: testosterone ratio [100].

In some studies, there have been associations of BPA with sexual functions, erectile functions, ejaculation, cryptorchidism and congenital genital malformations (due to the differentiation of Wolffian structures) in males [74]. In some comparative studies, it was concluded that BPA causes abnormalities in meiosis, spindle fibers and congenital defects in mice, pigs, cattle and humans [80,101].

Among the phthalates that cause damage to the male reproductive system, DEHP has been reported to alter the structure of Leydig and Sertoli cells, to inhibit testicular functions, to cause atrophy of the seminiferous tubule and to decrease testes weight and sperm production. There is also an association between the presence of DEHP and shortened anogenital distance, as well as with suppressed aromatase P450 enzyme expression (CYP19), which is the key factor for the conversion of androgens into estrogens [76], thus leading to masculinization of the brain. 

The findings in all these studies emphasize that additives for the production of microplastics disrupt the reproductive tissue functions.

## 8. Conclusions and Future Perspectives

The environmental pollution caused by plastic is due to accumulation in the oceans, atmosphere and soil of several synthetic polymers used for various human motives. Due to water, air and vegetables, these compounds can have effects on animals, including humans, by affecting their health and wellbeing. An increasing body of evidence suggests that farm animals can ingest plastics in various amounts, depending on environmental contamination. Potentially, these tiny particles and the additives used to enhance the efficacy and appearance of these plastics can cause damage to tissues and cellular systems due to their ability to activate various cascades of tissue functions, thus leading to inflammation, cytotoxicity, genotoxicity and immune toxicity in cells and tissues. Reproduction is particularly affected by these pollutants, as many of these can induce endocrine disruption. The phenotypic effects of these pollutants, when there have been studies conducted both in vivo or in vitro, are varied due to impaired fertility and hormone imbalance. The negative effects of plastic pollution on animal reproductive efficiency and the health of food-producing animals are not easy to ascertain due to the variety of confounding effects (nutrition, metabolism, productive level, management, etc.). The concern for the plastic hazard in the trophic chain and subsequent risk for animal and human health is growing among consumers and farmers. 

There remains the need to gain a better understanding of many of the components related to the information on this topic. Priority must be given to conducting further in vitro or in vivo studies and to better elucidate cellular and whole-body effects. Verifying the presence of the substances discussed in this article in animal products such as meat, milk and eggs also represents an important aspect for consumer safety. The scientific community should also direct efforts toward the identification of the best organic matrix to assess animal exposure (blood, urine, feces, milk and other tissues) and to identify the gold standards for analytical methods in animal feed, animal organic materials and animal-derived food products. 

Most importantly, there is a need for conscious behavior and for improved risk mitigation strategies through the reduction in exposure to substances that cause long-term harm to both humans and animals.

## Data Availability

Not applicable.
